# Influence of Biphasic Stimulation on Olfactory Ensheathing Cells for Neuroprosthetic Devices

**DOI:** 10.3389/fnins.2016.00432

**Published:** 2016-10-04

**Authors:** Rachelle T. Hassarati, L. John R. Foster, Rylie A. Green

**Affiliations:** ^1^Graduate School of Biomedical Engineering, University of New South Wales AustraliaSydney, NSW, Australia; ^2^Bio/Polymers Research Group, School of Biotechnology and Biomolecular Sciences, University of New South Wales AustraliaSydney, NSW, Australia

**Keywords:** PEDOT, olfactory ensheathing cells, electrical stimulation of nervous system, neural interfaces, living electrodes

## Abstract

The recent success of olfactory ensheathing cell (OEC) assisted regeneration of injured spinal cord has seen a rising interest in the use of these cells in tissue-engineered systems. Previously shown to support neural cell growth through glial scar tissue, OECs have the potential to assist neural network formation in living electrode systems to produce superior neuroprosthetic electrode surfaces. The following study sought to understand the influence of biphasic electrical stimulation (ES), inherent to bionic devices, on cell survival and function, with respect to conventional metallic and developmental conductive hydrogel (CH) coated electrodes. The CH utilized in this study was a biosynthetic hydrogel consisting of methacrylated poly(vinyl-alcohol) (PVA), heparin and gelatin through which poly(3,4-ethylenedioxythiophene) (PEDOT) was electropolymerised. OECs cultured on Pt and CH surfaces were subjected to biphasic ES. Image-based cytometry yielded little significant difference between the viability and cell cycle of OECs cultured on the stimulated and passive samples. The significantly lower voltages measured across the CH electrodes (147 ± 3 mV) compared to the Pt (317 ± 5 mV), had shown to influence a higher percentage of viable cells on CH (91–93%) compared to Pt (78–81%). To determine the functionality of these cells following electrical stimulation, OECs co-cultured with PC12 cells were found to support neural cell differentiation (an indirect measure of neurotrophic factor production) following ES.

## Introduction

Living-electrode systems are proposed as a means of encouraging better interactions between chronically implanted neuroprosthetic devices and target neural tissue (Green et al., 2013a; Aregueta-Robles et al., 2014). Utilizing living neural and glial cells at the surface, these tissue engineered electrodes have the potential to manipulate surrounding microenvironments and reduce natural foreign body and wound healing responses that often result in electrically resistive scar tissue formation about implanted devices. The neural and glial networks encapsulated within these hydrogel based systems have the potential to extend from the electrode interface and form synaptic connections with existing neural pathways in close proximity to the implanted device (Aregueta-Robles et al., 2014).

Schwann cells have been explored in prior literature as supportive glia in living-electrode systems (Ulises Aregueta-Robles et al., 2015). It has been shown that these cells can successfully facilitate neural regeneration through peripheral nerve guide conduits (Guénard et al., 1992; Ansselin et al., 1997) and support the formation of neural networks while encapsulated in 3D hydrogel systems (Suri and Schmidt, 2010). However, the tendency of these cells to segregate away from astrocytes in culture (Lakatos et al., 2000) implies that they are not the most ideal choice for a living-electrode system in the central nervous system (CNS), such as might be beneficial in brain machine interfaces (BMIs). Astrocytes are one of the major immune cells of the CNS and also support neuronal growth and development within the cortex. One of the first issues resulting from implantation of a neuroprosthetic device within the CNS is the proliferation and migration of astrocytes to the device interface (Joshua Burda and Michael Sofroniew, 2014). Thus, it is unlikely that a Schwann cell, sourced from the PNS would be a suitable cell to support neurons within living electrode systems.

Playing a supportive role in the olfactory system, the olfactory ensheathing cell (OEC) has been shown to assist neural growth through glial scars via the production of extracellular matrix (ECM) and the secretion of neurotrophic factors (Doucette, 1990; Woodhall et al., 2001). Lakatos et al. (2000) demonstrated the ability of these cells to migrate and integrate with the astrocytes in culture. OECs therefore have the potential to not only support the development of neural networks within the living-electrode system but could also assist the guidance of neurites through the glial scar. To determine the feasibility of utilizing this cell type in such a system the influence that electrode materials and relevant levels of electrical stimulation have on OEC viability and functionality must be assessed.

Charge-balanced biphasic stimulation is commonly utilized in neuroprosthetic devices to initiate the required ionic changes that trigger neural cell depolarization (Cogan, 2008). The alternating current direction associated with the biphasic pulse, allows for the reversal of chemical reactions which may have occurred at the electrode surface, however little is understood as to the impact this waveform will have on OECs at the electrode interface. The use of electrical charge to enact physiological changes in electroactive tissues has been used extensively since the introduction of the cardiac pacemakers in the 1920s (Lidwell, 1929). Studies of the impact of electrical charge on neural tissues, have focused almost exclusively on neurons and neuronal cell types, and few researchers have examined the impact of stimulation on the supporting glia. In 1997 McCreery et al. (1997) described the phenomenon of stimulus induced depression of neuronal excitability (SIDNE) when neural cells are continually activated. SIDNE was found to be induced in cells when electrostimulation frequency or voltages were too high. Electroporation and excitotoxicity are also potential issues with the continual activation of the cell, changing cell structure, and behavior (Ho and Mittal, 1996). Ultimately, electroporation or repeated exposure to voltages that alter cell membrane structures, beyond charge distribution, can lead to cell death (Pavlin et al., 2005). To overcome this issue, Green et al. (2012) developed a conductive hydrogel (CH) electrode coating by electrochemically depositing poly(3,4-ethylenedioxythiophene) (PEDOT) through a poly(vinyl-alcohol) (PVA)/heparin-methacrylate (PVA-hep) based hydrogel network. These coatings have been shown to produce surfaces that can deliver charge at substantially lower voltage in comparison to conventional Pt, as a result of the CH superior electrical and biological properties (Green et al., 2012). An advantage of these CH systems is that they can be easily modified during manufacture to incorporate biological molecules that induce tailored cell responses (Mario Cheong et al., 2014). Recent work by Hassarati et al. (2016) sort to determine a suitable CH electrode substrate for encouraging OEC attachment and proliferation. The incorporation of 1 wt.% gelatin within the PVA-heparin hydrogel was found to be sufficient to promote OEC proliferation on the electrode surface of the CH. This paper aims to examine the influence of clinically relevant levels of biphasic stimulation on the viability and functionality of OECs cultured on both CH and Pt electrode materials.

A comparison of the electrical characteristics of Pt and bioactive CH electrode surfaces have been recently reported (Hassarati et al., 2016). At low frequency stimulation (<1000 Hz), the CHs were found to significantly reduce electrical impedance compared to Pt, producing lower voltages at the electrode interface. Since clinically relevant electrical stimulation parameters are conventionally <1000 Hz (Shannon, 1983; Doucet et al., 2012; Guenther et al., 2012), it is hypothesized that the lower voltage charge transfer at the CH interface could improve cell survival when compared to Pt. As a result, CH coated electrodes may be able to support a higher viability of OECs at the neural interface of a bionic device that delivers electrical stimulation in comparison to Pt.

Like neural cells, OECs have been shown to proliferate upon being exposed to low levels of direct electrical stimulation, below 100 mV/mm (Qi et al., 2013). This paper proposed that biphasic stimulation at an appropriate level may similarly impact on OEC activity. Potentially this may yield shifts in the cell cycle, upregulating cell activities such as DNA replication (S-phase) and even mitosis, while reducing the percentage of cells in the rest phase. Biphasic stimulation may also up-regulate OEC production of the trophic factors associated with their support of neural cell growth and development. Specifically, OECs have been shown to produce BDNF and NGF in response to neural injury and regeneration. Prior work by Zorko et al. (2000) *in vivo* has shown that biphasic current pulses can create electrical fields that encourage neuronal regeneration. Specifically, having crushed the left and right radial nerves of the animal, a biphasic pulse (30 μA, 0.5 Hz) was passed through the left nerve for a period of 2 months. After the 2 month period, a significant increase in the electrical activity of the left (stimulated) musculus extensor was noted compared to the right (non-stimulated). This lead to the conclusion that the biphasic stimulus encouraged nerve regeneration. Similarly in recent studies by Qi et al. (2013) it was reported that direct electrical stimulation of OECs cultured on polypyrrole/chitosan films upregulated the production of neurotropic factors. As such it was expected that electrical stimulation can encourage OECs to produce NGF and BDNF.

Finally, to develop a living electrode structures, it is desirable to have both OECs and neuronal cells seeded on the electrode surface. A final aspect of this paper was to assess the supportive capability of OECs in co-culture with neural cells. PC12 cells derived from a pheochromocytoma in the adrenal medulla of rats and have long been used as a robust model in assessing neural interfacing biomaterials (Greene and Tischler, 1976). PC12s reversibly differentiate in response to the presence of NGF. Though the mechanism by which differentiation of these cells occurs is still unclear, it is known that they require good adhesion to the test sample surface (Lamour et al., 2015). *In vivo* it is the glial cells which provide the ECM that promoted cell adhesion and neurite outgrowth (Barros et al., 2011). In the absence of glial cells *in vitro*, it is common practice to coat sample surfaces with attachment proteins such as laminin, collagen and poly-L-lysine (PLL) to mediate PC12 attachment (Barros et al., 2011). In this study, OECs were cultured with PC12 cells on both Pt and CH coated electrodes, without additional ECM protein coatings, to assess the ability of OECs to support neural cell attachment and growth under electrical stimulation.

To assess the role of electrical stimulation in survival and proliferation of OECs on neural electrode materials, OECs were cultured on CH and Pt electrode surfaces. These electrodes were used to deliver two clinically relevant levels of biphasic stimulation (30–3000 μC/cm^2^; Cogan, 2008) based on vision and auditory neuroprosthetic devices. Image based cytometry was used to assess the influence of electrical stimulation on cell viability, apoptosis and cell cycle of the OECs relative to the electrode material type. The effects on the supportive functionality of these cells was assessed in co-cultures of a neurotrophin dependant cell line.

## Materials and methodology

### Electrode preparation

Pt discs (13 mm in diameter) were utilized as macroelectrodes. Of the 12 macroelectrodes used, six were prepared with a hydrogel hybrid of 17 wt.% PVA, 2 wt.% heparin, and 1 wt.% gelatin (Nafea et al., 2011) through which PEDOT was electropolymerised (PEDOT/17PVA-2Hep-1G). The fabrication process has been previously described by Hassarati et al. (2016). Briefly, a pre-layer of poly(3,4-ethylenedioxythiophene) (PEDOT) doped with para-toluenesulfonate (pTS) (PEDOT/pTS) was galvanostatically deposited on Pt at 1 mA/cm^2^ for 1 min. The hydrogel macromer solution was pipetted onto the sample, sandwiched under a coverslip then photocrosslinked by ultraviolet light (UV) (30 mW/cm^2^, 336 nm) for 180 s. The macromer solution comprised methacrylate modified PVA (17 wt.%, 13–23 kDa), heparin (2 wt.%, 17–19 kDa), and gelatin (1 wt.%, 50–100 kDa) dissolved in MilliQ water (70 wt.%, 18.2 MΩ/cm) and combined with the photo initiator [2-hydroxy-1-4-(hydroxyethoxy)phenyl]-2-methyl-1-propanone (1 wt.%, Irgacure 2959, Sigma-Aldrich). Following crosslinking of the hydrogel coating over the electrode, the surface was subsequently immersed in a 0.03 M EDOT aqueous solution. PEDOT was galvanostatically electropolymerised through the hydrogel layer at 0.5 mA/cm^2^ for 20 min. Finally, CH samples were soaked in DI water for 16 h to allow any excess macromer, EDOT monomer or other reagents to elute from the samples. The samples were then dried in a laminar-flow cabinet at RT.

### Electrical cell culture assembly

**Figure 1:**
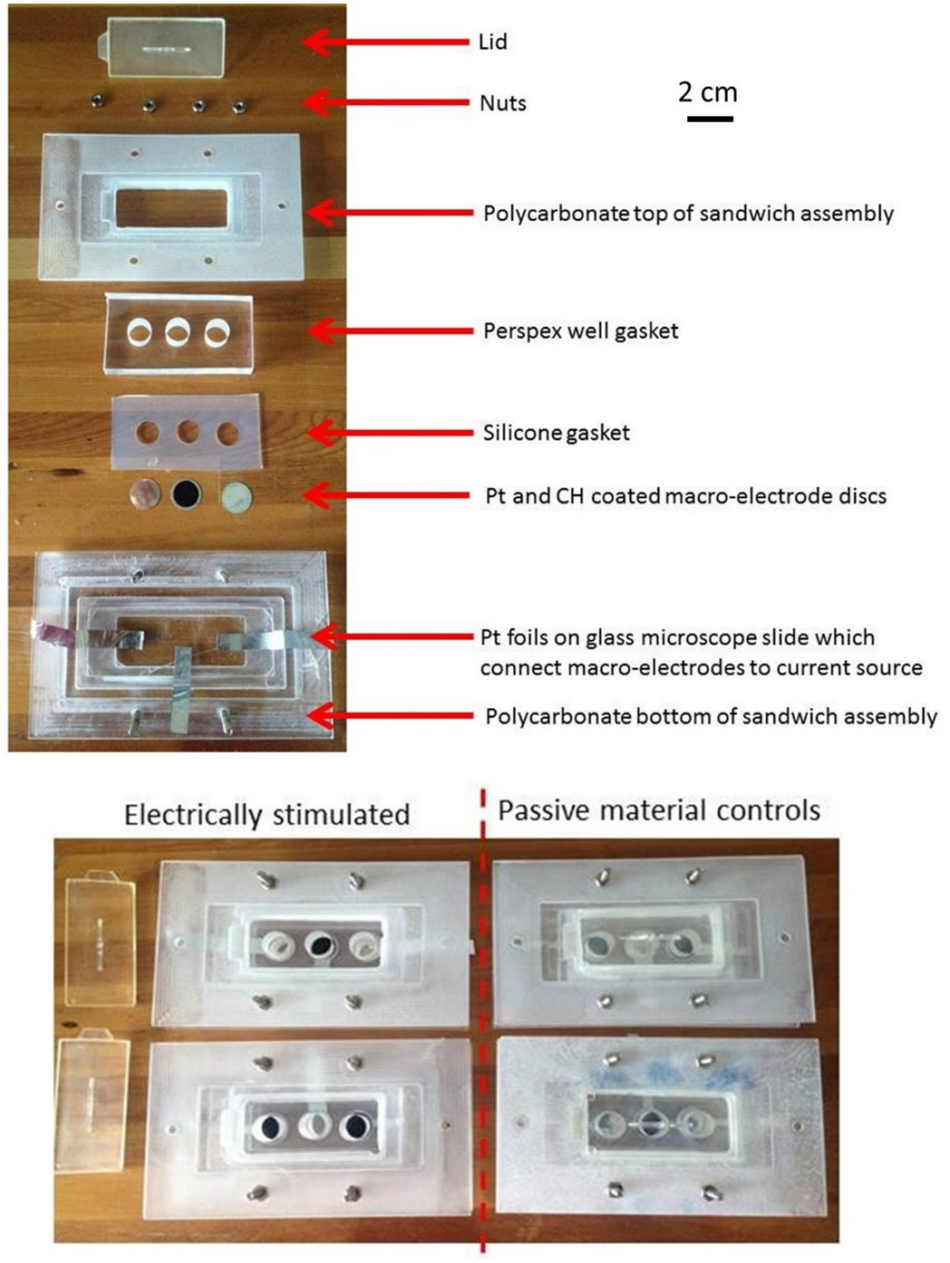
**The individual and assembled components of the electrical stimulation rigs with Pt and CH coated electrode substrates**.

Cell culture stimulation assemblies were manufactured in-house. The individual assembly components, Pt and CH coated electrode samples were disinfected under UV light (0.01 mW/cm^2^) for 1 h prior to assembly of the stimulation assembly in a laminar flow hood. Each assembly contained 3 electrically isolated wells (10 mm in diameter, Figure 1). The Pt and CH coated samples were assembled in an alternating pattern so as to reduce possible location bias. The assembled rigs were further disinfected under UV light for an additional hour.

### Electrical stimulation of glial cells

Murine OECs were extracted from 5-week-old Wistar rats (University of NSW ACEC number-06/53A) as described by Chan et al. (2011). The OECs were cultivated in Dulbecco's modified Eagle medium (DMEM, Gibco-Invitrogen, Australia) containing 10% fetal bovine serum (FBS), 250 units penicillin/250 μg mL^−1^ streptomycin and 1 μg mL^−1^ fungizone-amphotericin B (Gibco-Invitrogen, Australia). The OECs were incubated at 37°C with 5% CO_2_ and at 80% confluence harvested by trypsinization.

**Figure 2:**
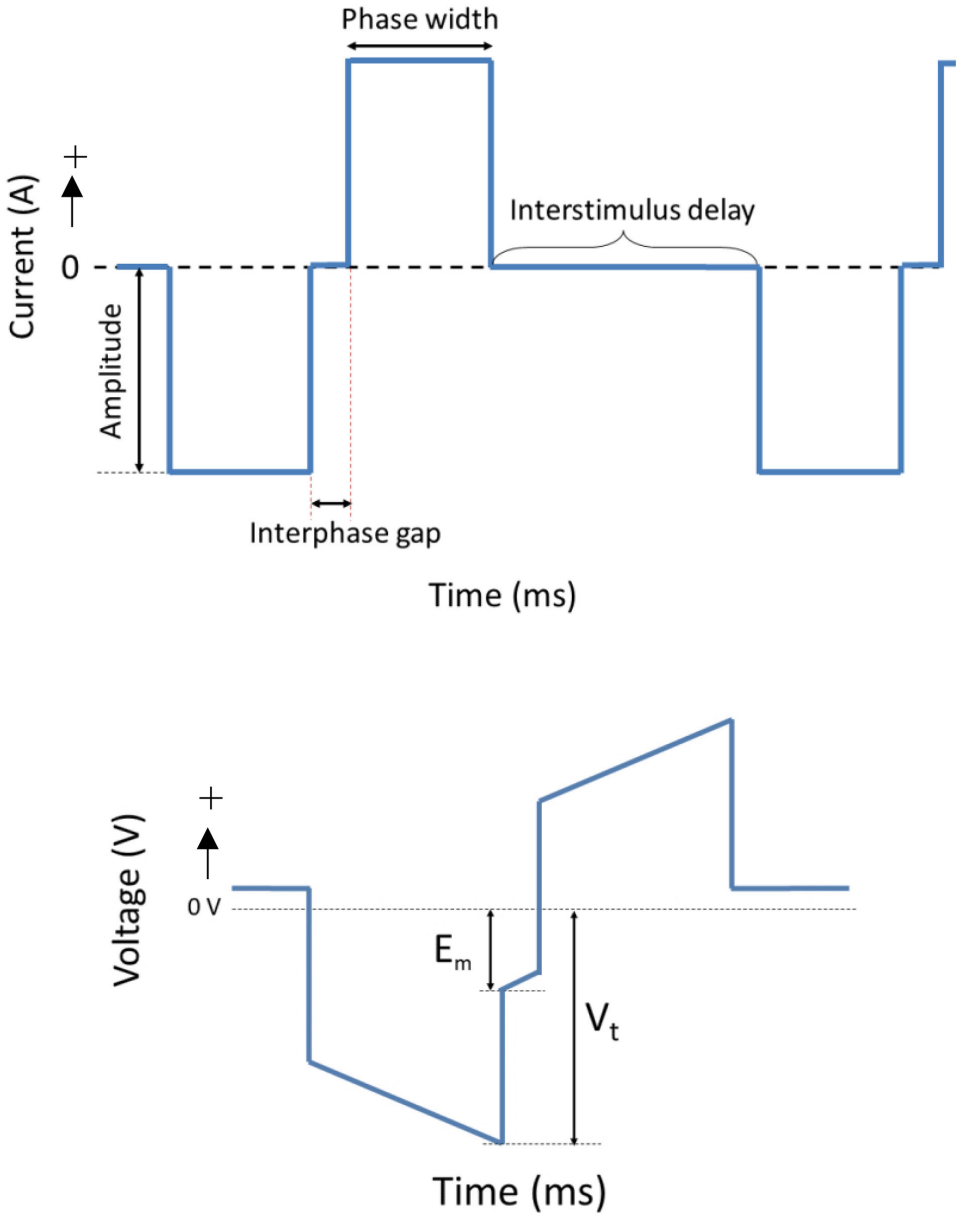
**A schematic of a cathodic first biphasic current and transient voltage waveforms**.

The OECs were plated on Pt and CH coated samples in Dulbecco's Modified Eagle's Medium (DMEM) containing 10% fetal bovine serum (FBS) and 1% penicillin-streptomycin (PS) at a density of 12,000 cells/cm^2^. Cells cultured at the same density on TCP (48-well plates) were used as cell health controls. At 48 h, Pt counter electrodes were introduced to the wells and a custom stimulator was used to apply a series of biphasic current pulses through half of the Pt and CH coated samples for 1 h. Stimulated OECs were subjected to one of two levels of biphasic stimulation with charge densities ranging from 30 μC/cm^2^ (Low Stim) to 3000 μC/cm^2^ (High Stim). The stimulation parameters used in this study were based on current density parameters commonly utilized by Cochlear Ltd (Dueck, Personal Communication) and in clinical trials for neuroprosthetic vision (Cogan, 2008). The amplitude of current delivery has been adjusted to account for electrode surface area, but charge density at the stimulating electrode is preserved. A schematic of a biphasic current waveform is shown in Figure 2 with a detailed description of the parameters utilized presented in Table 1. It should be noted that while these two charge densities are based in specific applications of implantable bionic devices, they are not necessarily considered clinically low or high levels of stimulation. To determine a “clinically” high or low level, one must know the arrangement and location of electrodes, including proximity to target tissue and the real surface area of the stimulating electrodes. These factors vary with electrode array choice (for example, planar vs. interpenetrating; Cogan, 2008; Dueck, Personal Communication). As a result the terms Low Stim and High Stim are nominal and used for ease of reference.

**Table 1:** **Electrical stimulation parameters used**.

**Electrical stimulation parameters**		
	**Low stim**	**High stim**
Amplitude	0.22 mA	2.2 mA
Pulse/phase width	0.1 ms	1 ms
Interphase gap	0.1 ms	0.1 ms
Interstimulus delay	6 ms	6 ms

During stimulation of cell cultures, the total voltage drop (V_t_) generated across the system as well as the residual voltage at the end of each phase (E_m_) was recorded. These are important electrode parameters that can be used to assess the electrochemical environment to which the cells are exposed. It is crucial that electrodes and the tissue with which they communicate are not exposed to residual voltages greater in magnitude than −800 mV. Continued exposure to higher levels of potential transients will alter the pH and pO_2_ balance of the tissue environment (Kadekaro et al., 1985). Additionally, these metrics are commonly used to assess the stability and efficacy of charge transfer from the electrical to the biological environment (Kadekaro et al., 1985; Huang et al., 2001).

After stimulation 2/3 of the media was refreshed for both stimulated and passive samples and the cell cycle control agents were added to the cell health controls samples. The cell health control samples are used to lock cell cultures in specific stages of the cell cycle and can then be used to benchmark the behavior of the cell population following electrical stimulation. Serum free DMEM (locking cells in the G0/G1 phase), 1 μg/ml Aphidicolin (S phase), and 2 μM/ml Nocodazole (G2/M phase) were each added to separate cultures, such that each phase had representation in triplicate. At 72 h post plating (24 h post stimulation) the conditioned media of the passive and stimulated samples was collected. Wells were rinsed twice with DPBS and the cells harvested using trypsin (10 min at 37°C). Full serum media was added to the cell suspension to inhibit the trypsin activity once cells were detached from the substrates.

### Cell cycle, viability, and apoptosis

The impact of biphasic stimulation on OEC viability, apoptosis and cell cycle was assessed by image-based cytometry as described by Daranarong et al. (2014). Briefly, the harvested cell samples were centrifuged (700 rpm, 5 min) and rinsed in DPBS. Cell pellets were resuspended in DPBS and divided into two aliquots, one for viability/apoptosis analysis and the other for cell cycle.

For viability/apoptosis, cells were washed in DPBS, centrifuged, and resuspended in 50 μl of Annexin V binding buffer and 2.5 μl Annexin V Alexa Fluor 488 (Thermo Fisher Scientific, USA). After incubation in a dark room (20 min at RT) cell samples were centrifuged (700 rpm, 5 min) and resuspended in 50 μl of Annexin V binding buffer to which 0.5 μl of propidium iodide (PI) staining solution (Thermo Fisher Scientific, USA) was added. Samples were incubated for a further 5 min before the Tali® Image-Based Cytometer (Thermo Fisher Scientific, USA) was used to perform suspension cell-based counting assays.

For cell cycle analysis, harvested OECs were washed in DPBS and subsequently fixed with ethanol (70%, −20°C, 16 h). Cells were then centrifuged (5000 rpm, 5 min), washed in DPBS and centrifuged again. The resulting pellets were resuspended in 50 μl of cell cycle premixed reagent consisting of PI, RNase A, and Triton X-100 (Thermo Fisher Scientific, USA). After an incubation period of 15 min in the dark, 25 μl of each cell suspension sample was analyzed using the Tali image-based cytometer.

### OEC support of neural cells

The Pt and CH electrodes were prepared and placed in assemblies as described in Section Electrical Cell Culture Assembly. PC12 cells (20,000 cells/cm^2^) and co-cultures of PC12s (20,000 cells/cm^2^) with OECs (10,000 cells/cm^2^) were plated in DMEM/Ham's F12 (Sigma) supplemented with 10% FBS and 1% PS on sample surfaces in triplicate. Cells plated on passive (unstimulated) Pt and CH electrodes were used as controls. It should be noted cells were plated on electrode samples which had no prior coating of biological attachment proteins (such as laminin or PLL) so to determine the influence of OECs on PC12 attachment and differentiation. After 48 h incubation (37°C, 5% CO_2_), a series of biphasic stimulation pulses were passed through the electrodes for 1 h using the High Stim parameters presented in Table 1. Samples were maintained at 37°C, 5% CO_2_ and 100% humidity during the period of stimulation. After electrical stimulation, the media was refreshed for both the stimulated and passive samples. At 72 h incubation (24 h post stimulation) cell samples were rinsed twice with DPBS and fixed in 4% paraformaldehyde in DPBS.

Immunocytochemistry was used to detect OEC and PC12 attachment and PC12 neurite outgrowth on the electrode samples. Fixed samples were rinsed three times with DPBS and incubated with permeabilisation buffer (0.5% Triton x-100 in DPBS, 30 min, RT). After rinsing three times with DPBS, samples were then incubated with blocking buffer solution (0.2% gelatin, 0.1% Triton x-100, 2% bovine serum albumin) for 30 min. To visualize PC12 neurite outgrowth, samples were incubated with the primary antibody, anti-mouse: anti-βIII-tubulin (1:200 in blocking buffer solution, 16 h at 2°C). Additional washing was undertaken with blocking buffer solution, and then the secondary antibody IgG Dylight-488 anti-Mouse (1:500) and the nucleus counterstain bisbenzimide (Hoechst 33342; Sigma, 1:1000) were gently added to the samples. After incubation (2 h at 2°C) samples were rinsed with DPBS and imaged using epifluorescence microscopy (x200, Olympus, IX83 System). PC12 cell adhesion and neurite outgrowth was assessed using the semi-automated plugin Simple Neurite Tracer in the Fiji/ImageJ software. Neurite lengths were traced from the tip of the neurite to the junction between the cell body and neurite base as described by Schmidt et al. (1997).

## Results

Electrical stimulation of cell culture systems was employed to assess the capacity of CH (PEDOT/17PVA-2Hep-1G) coated electrodes to support OECs functionality under application specific conditions. Explicitly this included delivery of clinically relevant levels of electrical stimulation used in implantable bionic devices in comparison to conventional Pt electrodes.

### Voltage across the culture system

**Figure 3:**
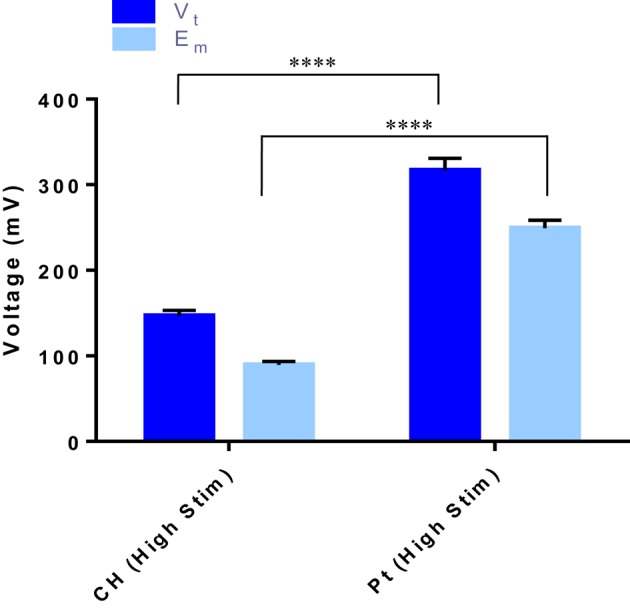
**The total voltage (V_**t**_) and the residual voltage (E_**m**_) at the of the cathodic phase for both the CH and Pt electrode systems**. Error bars represent one standard deviation. (*t*-test, ^****^significant difference, *p* ≤ 0.0001, *n* = 3).

When OEC cultures were placed under electrical stimulation, the potential transient of the biphasic stimuli was measured. These metrics are an important tool in establishing the electrochemical environment to which the cells are exposed. Presented in Figure 3 is the average total voltage measured at the end of the cathodic phase (V_t_) for each of the electrode systems, and the residual voltage across the system at the end of the phase (E_m_). For the same current input, there was a significantly lower average voltage recorded across the CH samples (147 ± 3 mV) compared to the voltage across the Pt samples (317 ± 5 mV).

### Viability and apoptosis

**Figure 4:**
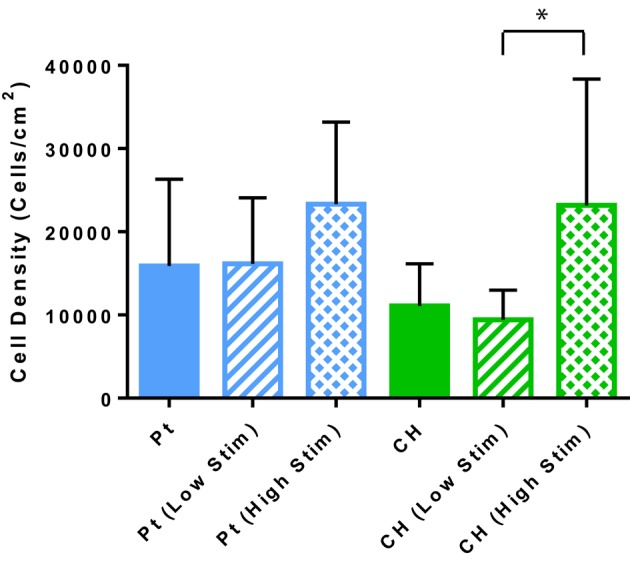
**The estimated OEC density on each sample as determined by the cell concentration measured with the Tali**. Error bars represent the standard error of the mean. (*t*-test, ^*^significant difference, *p* < 0.05, *N* = 3).

Cell survival and damage to cells imparted by electrical stimulation is also a critical factor to the longevity of a cell loaded electrode. In Figure 4 the OEC densities on the passive and stimulated samples are presented. These cell counts were generated by the image-based cytometer during viability and apoptosis analysis. A higher average number of OECs were harvested from the Pt electrodes compared to the CH coated electrodes for each tested variable. This correlates with prior studies where cell density was assessed via microscopy (Hassarati et al., 2016). These values were not statistically significant, but may warrant investigation in future studies as cell attachment and motility can substantially impact cell proliferation and function as supportive glia. Overall, biphasic stimulation had minimal influence on the number of cells harvested from the samples, however, higher levels of stimulation appeared to encourage cell proliferation, with a greater effect observed for the CH coated electrodes.

**Figure 5:**
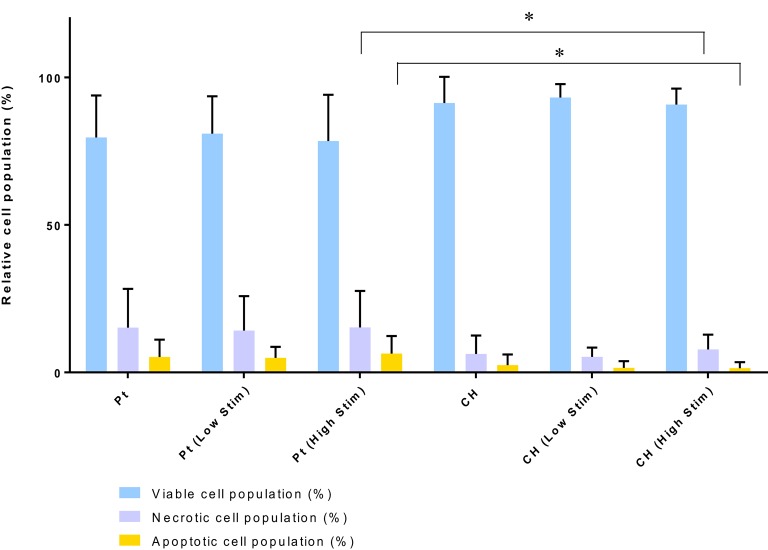
**Relative viable, necrotic, and apoptotic cell populations**. Error bars represent standard deviation. (Tukey, Two-way ANOVA multiple comparisons test, ^*^p < 0.05, *n* = 3).

The relative health of the OEC populations cultured on the passive and stimulated samples are presented in Figure 5. While there were fewer OECs harvested from the CH electrodes under passive and Low Stim conditions, these cells appeared to have a higher average percentage of living cells (91–93%) compared to the Pt (78–81%). Consequently, fewer apoptotic and necrotic cells were found on the CH samples compared to those cultured on Pt. It is expected that the variability in data for OEC viability on Pt samples was related to their strong adherence to the metallic substrate. To adequately remove cells for analysis, repeat washes were required and it was found that cell number and viability data had wider variability on this material. As a result, statistically significant differences between most of these samples were not observed. However, for the high level of biphasic stimulation applied to the Pt electrodes there was a significant increase in the percentage of apoptotic and necrotic cells compared to the OECs cultured on the CH electrodes. While impacts on cell number and viability have been observed, the majority of the cell population subjected to biphasic stimulation at these levels have survived with no significant difference between passive (unstimulated) and active (stimulated) electrodes of the same material type.

### Cell cycle

**Figure 6:**
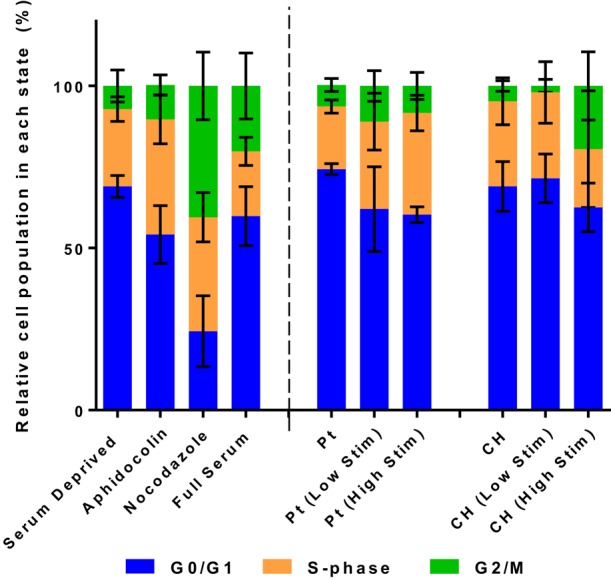
**The relative distribution of OECs cultured on Pt and CH electrodes found to be in the cell cycle phases G0/G1, S, and G2/M**. Cell health controls in serum deprived media and full serum media with the addition of aphidicolin (S phase control) and nocodazole (G2/M phase control). Error bars represent the standard error of the mean.

The relative population of OECs in each phase of the cell division cycle are presented in Figures 4, 6. Samples were compared to TCP controls, a substrate on which the health of OECs has been investigated in prior studies (Daranarong et al., 2014). These controls were established by placing the cells in environmental conditions that have been shown to arrest cell development at a particular stage of the cell cycle. Cell cycle controls are presented on the left side of the dotted line in Figure 6. The OECs cultured on the Pt electrodes were shown to have a significant shift into the S phase following biphasic stimulation (*p* < 0.05, for passive vs. Low Stim and passive vs. High Stim). While lower levels of biphasic stimulation had little influence on cell cycle of the OECs cultured on CHs, the higher levels of stimulation pushed OECs into the DNA replication and mitosis phases (*p* < 0.05, for passive CH vs. High Stim). This data concurs with the viability assay, suggesting electrical stimulation may promote cell proliferation.

### Supportive capacity of OECs

**Figure 7:**
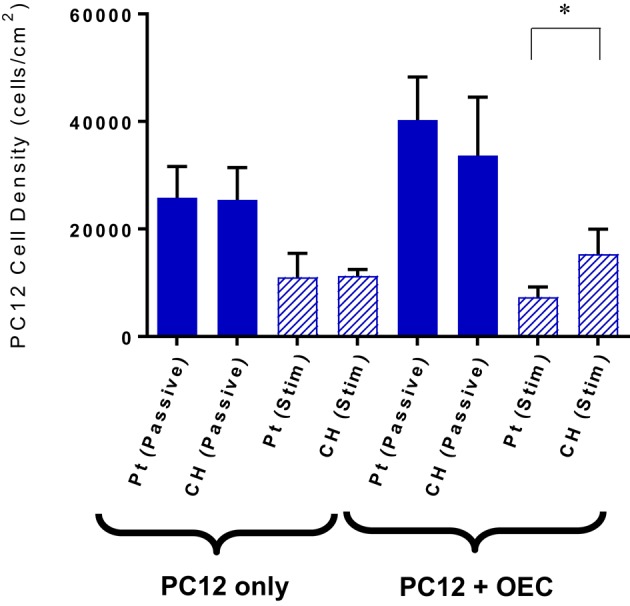
**Quantification of PC12 cell attachment when cultures with and without OECs on passive and electrically stimulated Pt and CH electrodes**. Statistical analysis was in the form of Tukey one-way ANOVA, ^*^p < 0.05. Error bars represent the standard error of the mean.

Under co-culture conditions the cell attachment and differentiation of the PC12s were considered the most important indicators of OEC function. Figure 7 shows the PC12 cell and neurite density values for the cells cultured with or without OECs on passive or electrically stimulated Pt and CH coated electrodes. The presence of OECs in the culture had a marginal influence on the proliferation of the PC12 cells, which was unexpected as they were cultured under differentiation conditions where proliferation should not occur. It can also be seen that the number of PC12s was greater on the CH when cultured with OECs and subjected to stimulation, than that for the Pt substrate.

**Figure 8:**
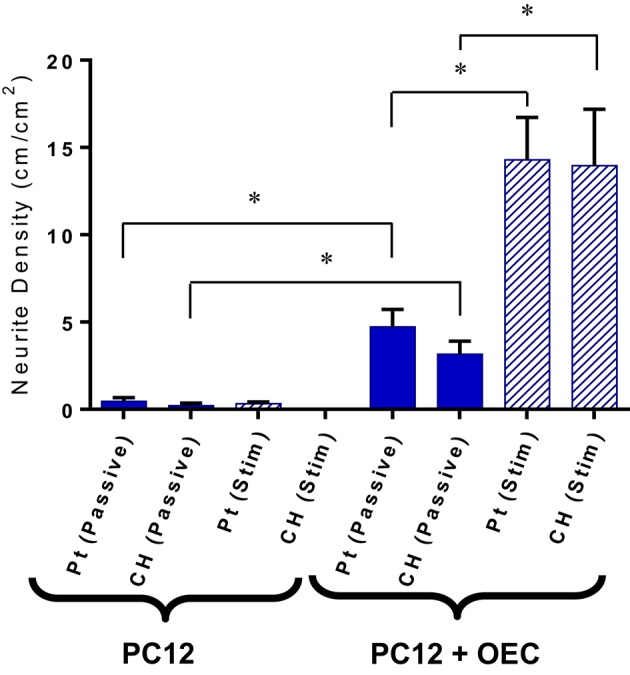
**Quantification of neurite outgrowth density for PC12 cells cultures with and without OECs on passive and electrically stimulated Pt and CH electrodes**. Statistical analysis was in the form of Tukey one-way ANOVA. ^*^Shows significant differences for *p* < 0.05. Error bars represent the standard error of the mean.

Upon application of stimulation the neurite outgrowth from PC12s cultured with OECs was increased (Figure 8). It should be noted that while OECs clearly provided support for PC12s even in under electrically passive conditions, the combination of both OECs and stimulation produced the most substantial differentiation and growth of neural processes. This suggests that despite the difficulty in quantifying neurotrophic output of OECs, they are in fact producing neurotrophins, and in particular NGF, which was not added to the cultures, but is critical for PC12 differentiation.

**Figure 9:**
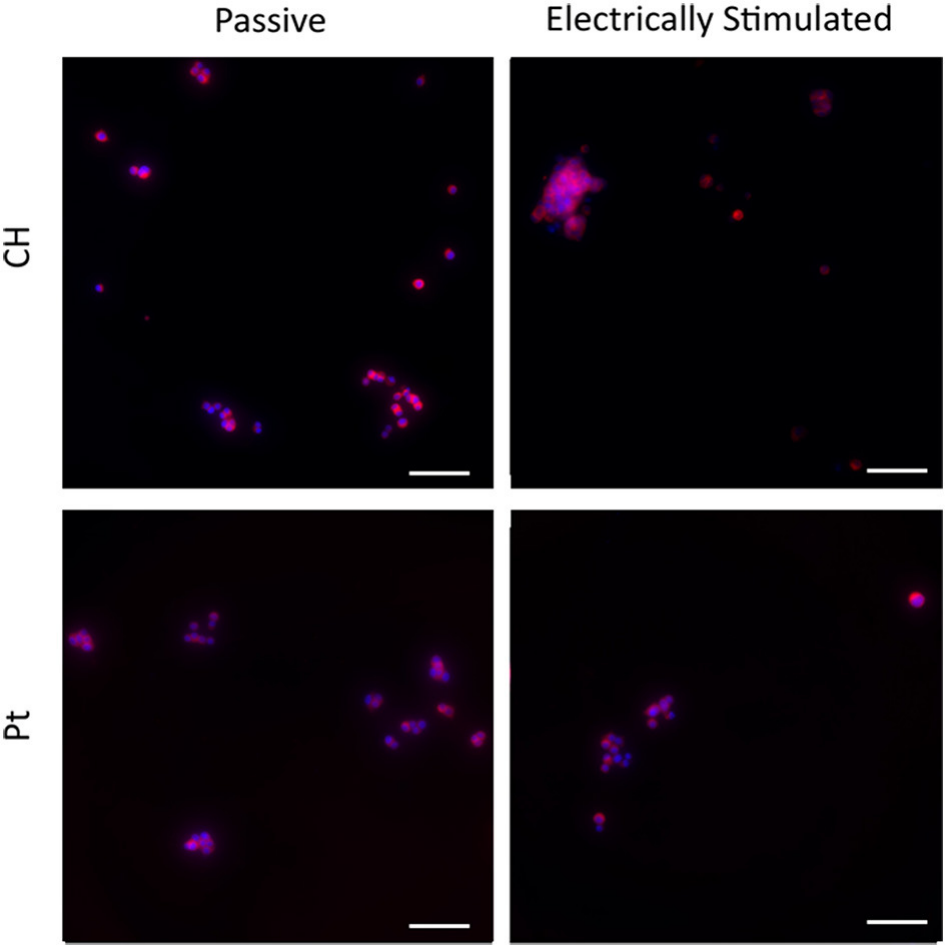
**Magnified immunofluorescence images of PC12 cultures on passive and electrically stimulated Pt and CH substrates**. Nuclei were stained with Hoechst 33,342 (blue) and PC12 cell bodies and neurites stained with anti-βIII-tubulin (red). Scale bars = 150 μm.

**Figure 10:**
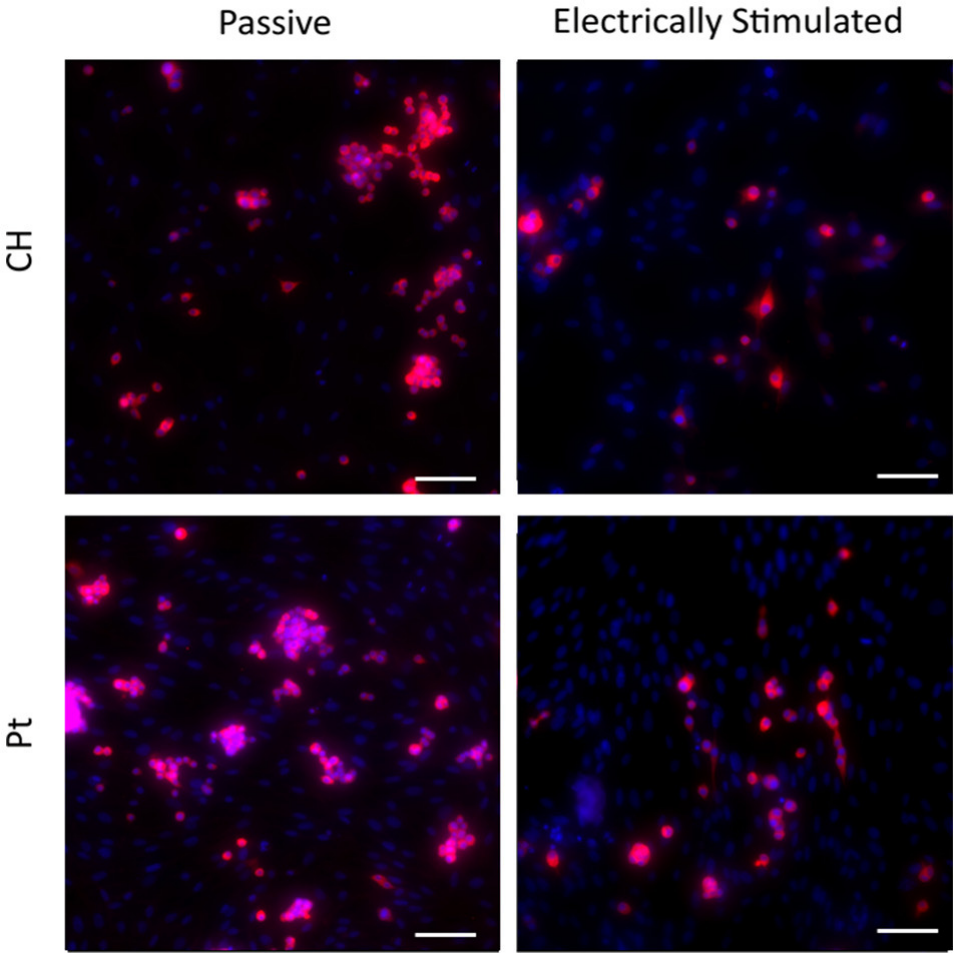
**Magnified immunofluorescence images of PC12 and OEC co-cultures on passive and electrically stimulated Pt and CH substrates (200 × magnification)**. Nuclei of both cell types were stained with Hoechst 33,342 (blue) and PC12 only cell bodies and neurites stained with anti-βIII-tubulin (red). Scale bars = 150 μm.

Typical images are presented in Figures 9, 10. Figure 9 demonstrates how cultures of PC12s alone have minimal attachment to electrode surfaces and as a result poor neurite outgrowth regardless of the material or electrical state. Figure 10 illustrates how OECs clearly support better PC12 morphologies, with PC12s observed to have extended cytoplasm and higher neurite outgrowth densities. It should also be noted neurite outgrowth is not extensive, but this is expected after relatively short time following plating.

## Discussion

The influence of electrode materials on OEC viability and functionality under biphasic stimulation was examined. Cells cultured on CH and Pt surfaces were subjected to two levels of clinically relevant biphasic stimulation and the cell health, cell cycle and supportive capacity of the OECs were examined. The levels of biphasic stimulation applied in these studies had minimal influence on the number of cells present on the electrode surfaces, though higher levels of stimulation appeared to begin encouraging OEC proliferation, but may have reduced attachment of PC12s. This observation was reflected in cell cycle studies which suggest that electrical stimulation encourages DNA replication and mitosis in OECs, both important elements of proliferation. While quantification of biochemical factors generated by OECs was not possible using the available equipment, an indirect assay demonstrated that growth factors produced by the OECs were sufficient to support PC12 differentiation into neural phenotypes.

Biphasic stimulation at these levels had no negative impact on the cell health of the OECs, with CH coated electrodes having slightly higher percentage of living cells compared to the Pt electrodes. This could be due to the significantly smaller voltages occurring across the CH electrodes, which were less than half that of the Pt under the higher level of stimulation (147 vs. 317 mV). Similar results were observed by Green et al. (2013b) when investigating the performance of conductive polymer (CP) electrodes *in vivo*. In this prior study it was found that PEDOT coated microelectrodes experienced a potential transient during charge transfer that was roughly half that of the Pt control (1.5 vs. 3.3 V). While these microelectrodes were considerably smaller, and as a result the voltage was an order of magnitude higher, the same relative relationship persists. In this study, the CH materials, having a lower impedance and improved charge transfer, may mediate the effects of the electrical stimulus on the OECs, resulting in better ratios of living to dead OECs cultured on the CH surfaces compared to the Pt.

The lower level of biphasic stimulation had little influence on cell cycle of the OECs cultured on the CH electrodes compared to their passive counterparts. However, higher levels of stimulation through the CH electrodes appeared to push OECs into the DNA replication/mitosis phases which were supported by the cell density data. The number of cells harvested from the CH electrodes, and measured by cell cytometry, was close to those previously published (Hassarati et al., 2016). Hassarati et al. (2016) reported a significantly greater number of OECs attached to the Pt compared to the CH samples yet the number of cells harvested from the stimulation study were not statistically dissimilar. Despite multiple DPBS washes, trypsinisation and a media rinse it is possible that not all cells had detached from the Pt surface. This could be due to cells being more strongly attached to the Pt electrodes compared to the CHs. Additionally, it is possible that the OECs produced a greater amount of ECM to adhere to the inorganic Pt surface compared to the CH in which the biological attachment molecule gelatin was incorporated.

Electrical stimulation has long been used as a tool for neural regeneration as it encourages neural differentiation and neurite outgrowth (Patel and Poo, 1982). While the mechanisms by which electrical stimulation trigger neurite outgrowth are still unclear, it has been hypothesized that the resulting protein absorption to implanted conductive substrates assist in neurite extension (Kotwal and Schmidt, 2001). The PC12s cultured with OECs on both passive and stimulated electrode materials showed an increase in neurite density compared to PC12s cultured alone. This correlates with studies performed by Feng et al. (2008) who explored the effects of OEC conditioned media on the neurite outgrowth of neuroblastic PC12 cells. In these preliminary studies it was shown that PC12s cultured with the conditioned media had a significant increase in neurite outgrowth compared to the standard DMEM, high serum control. Schmidt et al. (1997) also observed on application of 100 mV across CP films, that the neurite lengths of cultured PC12 cells had almost doubled. On application of the biphasic stimulus in this study, while PC12 cell density decreased, the neurite density of the PC12 cells cultured with OECs almost tripled in value compared to their passive counterparts. Since the percentage of viable OECs did not differ significantly between passive and stimulated samples, the increase in neurite density suggests that biphasic stimulation is encouraging the OECs to assist in the differentiation of the PC12 cells. This could be by either increased secretion of growth factor proteins or greater production of ECM. By considering the impact of electrical stimulation on OECs alone, the latter scenario is the most likely. While electrical stimulation did not show increased output of NGF, it was shown to shift the distribution of cells out of the resting state and into active DNA replication and mitosis. As such, it is probable that ECM production would also be increased in line with this increased cell activity. Further studies are required to investigate the relationship between ECM and electrical stimulation of glia, with a focus on the threshold level of electrical stimulation required to impart changes to cell proliferation and neural differentiation.

Another factor to consider when comparing the neural cell growth on these electrode materials is their hydrophilicity. While Pt is clearly hydrophobic, CHs are strongly hydrophilic (Green et al., 2012). This difference in surface chemistry will substantially impact on protein and hence cellular interactions. The low fouling nature of the CH has been previously shown to reduce impedance of electrodes when submerged in protein based artificial perilymph solutions (Hassarati et al., 2014). This could also explain why there may have been less neurite outgrowth for PC12s cultured alone on CHs samples, as there is expected to be less protein available at the surface during electrical stimulation. With electrically mediated extension of neurites being reliant on protein rearrangement, the lack of protein available at this surface will considerably impede neurite growth.

A significant finding of these studies is that the OECs cultured on novel CH electrode materials do produce growth factors, and in particular NGF, that is shown to promote differentiation of PC12s. In prior studies, Green (2009) explored the effect of NGF concentrations on the PC12 neurite outgrowth. PC12 cells were cultured on laminin coated TCP plates and supporting media was supplemented with NGF concentrations ranging from 0 to 100 ng/ml. With no NGF available, no neurites where observed. On the addition of 10 ng/ml of NGF to the culture media, the neurite density increased to ~30 cm/cm^2^ (Green, 2009). As the NGF concentration increased, so too did the neurite density and number of attached PC12 cells. In this study, the media was not supplemented with any NGF, and as a result all PC12 neurite extension was the result of OEC produced factors. The PC12s cultured with OECs and subjected to biphasic stimulation generated an average neurite density close to 14 cm/cm^2^. This suggests that the OECs subjected to biphasic stimulation were producing NGF at a concentrations under 10 ng/ml, and if a linear behavior persists, close to 5 ng/ml. To increase the NGF output several approaches could be used, including seeding of a greater number of OECs, tuning the electrical stimulation to increase proliferation and protein production, or alternately functionalizing the CH with forskolin to promote increased growth factor output.

## Conclusion

The studies performed in this paper sought to determine the influence of electrical stimulation on OEC functionality cultured on Pt and CH electrode substrates for the eventual incorporation into living electrode systems. Clinically relevant biphasic stimulation was shown to have a negligible impact on cell viability, while shifting the cell cycle of OECs cultured on either the Pt or CH coated electrodes into a more proliferative state. The lower voltages measured across the CH systems compared to the Pt showed the ability of these materials to transduce high stimulation currents with less impact on cell health, which was reflected in the higher percentages of viable cells on CH compared to Pt electrodes. Co-cultures of PC12s and OECs suggest that NGF is produced by the OECs and these glial cells do support the differentiation of neural cells under biphasic stimulation. Having shown OECs to be capable of supporting neuron differentiation under biphasic stimulation on CH electrodes, a method of incorporating these cells within the living-electrode system must be found. Future work will seek to characterize the influence which encapsulating OECs within a degradable CH electrode system has on OECs viability and functionality.

## Author contributions

RH: Made the acquisition, analysis and interpretation of data for the work; and drafted the work gave final approval of the version to be published and agrees to be accountable for all aspects of the work in ensuring that questions related to the accuracy or integrity of any part of the work are appropriately investigated and resolved. LF and RG: Assisted in interpretation of data for the work; revised it critically for important intellectual content gave final approval of the version to be published and agrees to be accountable for all aspects of the work in ensuring that questions related to the accuracy or integrity of any part of the work are appropriately investigated and resolved.

### Conflict of interest statement

The authors declare that the research was conducted in the absence of any commercial or financial relationships that could be construed as a potential conflict of interest.
